# HOme-based self-sampling for cervical cancer prevention education among women living with HIV in Ghana (HOPE-inG): study protocol for a cluster-randomized type 2 hybrid effectiveness implementation trial

**DOI:** 10.1186/s12889-026-26505-2

**Published:** 2026-03-27

**Authors:** Matthew Asare, Nadia Adjoa Sam-Agudu, Obed Cudjoe, Emily Frueh, Karen Awura-Adjoa Ronke Coker, Alex Boadi Dankyi, Sebastian Ken-Amoah, Patrick Kafui Akakpo, Nancy Innocentia Ebu Enyan, Rodney X. Sturdivant, Emmanuel Ekow Asmah, Gloria F. Nuer-Allornuvor, Dorcas Obiri-Yeboah

**Affiliations:** 1 Department of Public Health, Robbins College of Health and Human Sciences, Baylor University, Bear Place, PO Box 1, Waco, TX USA; 2 Global Pediatrics Program, Department of Pediatrics, University of Minnesota Medical School, 717 Delaware Street SE, Minneapolis, MN 55455 USA; 3 Division of Pediatric Infectious Diseases, Department of Pediatrics, University of Minnesota Medical School, 2450 Riverside Avenue S, AO-103, Minneapolis, MN 55454 USA; 4 International Research Center of Excellence, Institute of Human Virology Nigeria, Plot 62 Emeritus Umaru Shehu Way, Abuja, Nigeria; 5 Department of Paediatrics and Child Health, School of Medical Sciences, University of Cape Coast, University Post Office, Cape Coast, Ghana; 6 Department of Medical Laboratory Science, School of Allied Health Sciences, College of Health and Allied Sciences, University of Cape Coast, University Post Office, Cape Coast, Ghana; 7 Robbins College of Health and Human Sciences, Baylor University, PO Box 1 Bear Place, Waco, TX USA; 8 Directorate of Research, Innovation and Consultancy, University of Cape Coast, University Post Office, Cape Coast, Ghana; 9 Department of Obstetrics and Gynecology, School of Medical Sciences, College of Health and Allied Sciences, University of Cape Coast, University Post Office, Cape Coast, Ghana; 10 Department of Pathology, School of Medical Sciences, University of Cape Coast, University Post Office, Cape Coast, Ghana; 11 Department of Public Health Nursing, School of Nursing and Midwifery, College of Health and Allied Sciences, University of Cape Coast, University Post Office, Cape Coast, Ghana; 12 Department of Statistical Science, College of Arts & Sciences, Baylor University, 1 Bear Place, 97344, Waco, TX 76798 USA; 13 Department of Data Science and Economic Policy, School of Economics, College of Humanities and Legal Studies, University of Cape Coast, University Post Office, Cape Coast, Ghana; 14 Department of Obstetrics and Gynecology, Cape Coast Teaching Hospital, P. O. Box CT 1363, Cape Coast, Ghana; 15 Department of Microbiology and Immunology, School of Medical Sciences, College of Health and Allied Sciences, University of Cape Coast, University Post Office,, Cape Coast, Ghana

**Keywords:** Women, HIV, HPV, 3R model, Cervical cancer, Ghana, HIV infections, Health education

## Abstract

**Background:**

Women living with HIV (WLWH) are six times more likely to develop cervical cancer (CC) than women without HIV, underscoring the urgent need for early and frequent screening. In Ghana, however, screening uptake among WLWH remains critically low and no national screening program exists. The HOPE (HOme-based self-sampling Prevention Education) toolkit has proven effective in increasing screening uptake. HOPE-inG (HOPE 2.0 in Ghana) aims to adapt the HOPE toolkits and develop implementation support strategies (ISS) to enhance the uptake and sustainment of cervical screening within HIV care services.

**Methods:**

This study will be conducted in four HIV clinics in Ghana’s Central Region using a three-phase design.

Preparation Phase (Year 1): Use mixed methods, including nominal group techniques with 40–60 stakeholders (WLWH, healthcare providers, policymakers), to prioritize ISS and refine HOPE into HOPE 2.0 or HOPE-inG.

Implementation Phase (Years 2–3): Conduct a hybrid type 2 cluster-randomized controlled trial, comparing HOPE 2.0 + ISS (intervention group) versus HOPE 2.0 alone (control group). A total of 1,152 WLWH and 80 health providers will be enrolled.

Sustainment Phase (Years 3–5): Evaluate the long-term sustainment of HOPE 2.0 and ISS using quantitative and qualitative methods.

Primary outcomes include patient-level screening and treatment uptake (effectiveness) and provider-level implementation metrics (adoption, cost, fidelity, penetration and sustainability). Data sources include surveys, interviews, electronic tracking and fidelity checklists at multiple organizational levels. Cost analyses will employ micro-costing methods, and fidelity will be monitored through structured observations and log sheets.

**Discussion:**

HOPE-inG is designed to develop scalable, sustainable strategies for improving cervical screening and care among WLWH in Ghana. By embedding evidence-based screening tools within HIV care services and supporting providers with targeted ISS, this study seeks to bridge critical gaps in cervical cancer prevention. Findings will provide evidence to inform national policy and guide the broader implementation of patient-driven and community-based screening programs in other low-resource settings, ultimately improving health outcomes and reducing disparities for WLWH.

**Trial registration:**

ClinicalTrials.gov NCT06800664. Registered on 29 January 2025.

**Supplementary Information:**

The online version contains supplementary material available at 10.1186/s12889-026-26505-2.

## Background

Women living with HIV (WLWH) have a six-fold higher risk of developing cervical cancer (CC) than women without HIV [1]. The World Health Organization (WHO) recommends nucleic acid-based human papillomavirus (HPV) testing for cervical screening (CS) among WLWH beginning at age 25 years, and every 3 to 5 years thereafter [2]. Early detection and prompt treatment of precancerous lesions of the cervix are crucial to preventing CC in this high-risk group CC [3–5]. However, women in low and middle-income countries (LMICs) face significant challenges in accessing cervical screening and treatment services.

Ghana, with an HIV prevalence of 1.7% in the general population, has a feminized epidemic: approximately 64% of the estimated 330,000 people living with HIV are women, aged 15 years and above [6–9]. However, Ghana lacks a national cervical screening program [10–13] and CC prevention programs are not commonly promoted. Screening rates are extremely low, with only 2.7% of eligible women ever screened [14]. Among those who test positive for high-risk HPV, many are lost to follow-up [13, 15, 16]. There is no evidence that WLWH are screened or treated at higher rates than women without HIV. These gaps are particularly consequential for the estimated 69,000 Ghanaian WLWH, who remain at increased risk [17]. In Ghana and other low- and middle-income countries, barriers to attaining the WHO goal of at least 70% cervical screening rates and subsequent treatment [18] include limited healthcare funding, inequitable distribution of services, and limited capacity of healthcare workers [12, 16, 19, 20]. Other barriers include misconceptions, cultural beliefs, and societal stigmatization, fear of pain, prohibitive costs, privacy concerns, and low prioritization of cancer screening [21, 22].

To address some of these challenges, our research team developed and tested a HOme-based self-sampling cervical cancer Prevention Education (HOPE) toolkit [23]. The HOPE toolkit consisted of HPV self-sampling, the 3R (Reframing, Reprioritizing, and Reforming) communication model [23–26] and patient navigator support. The current study is an extension of the HOPE initiative to promote cervical screening among women WLWH in Ghana (hereafter referred to as HOPE-inG or HOPE 2.0). HOPE-inG retains the three core components of the original HOPE intervention and introduces a fourth component: implementation support strategies (ISS), which we define as implementation strategies most relevant to implementation support, which is concerned with moving (implementation) research into (implementation) practice. ISS facilitate health system adoption and sustainment and function most directly within the implementation setting and at the interface between implementation and policymaking entities.

### Study aims

Although HOPE components have been rigorously tested and found impactful in our Ghanaian study setting, the absence of implementation strategies and a structured implementation plan has limited their translation into routine clinical practice. An implementation science approach guides the development of implementation strategies for adopting and translating this next phase of HOPE 2.0 into routine health practice in Ghana. Our study aims are as follows:

1. To develop a culturally appropriate, evidence-based implementation plan and provider training materials to support the effective delivery of the HOPE 2.0 intervention within Ghana’s health system.
2. To evaluate the effectiveness of the HOPE 2.0 intervention and its implementation strategy through a hybrid type 2 implementation-effectiveness trial.
3. To assess the impact of the implementation plan on the sustainment of the HOPE intervention at participating HIV clinics.

## Methods

This HOPE-inG study is a two-arm, cluster-randomized type 2 hybrid effectiveness implementation trial.

### The HOPE intervention

The evidence-based intervention (EBI) for our study is HOPE’s three-component combination intervention: a cervical screening self-sampling kit, the 3R communication model, and patient navigator support.

#### Evidence-based HPV self-sampling

HPV self-sampling (HPVSS) is a feasible and effective cervical screening strategy [27–30]. It involves women collecting their own vaginal samples, which are then sent to health facilities or laboratories for analysis to detect HPV strains. Compared with clinician-collected samples, HPVSS can help overcome both structural and individual-level barriers to cervical screening and thus increase participation [31]. Meta-analyses indicate that HPVSS is comparable in sensitivity to clinician-collected PCR samples and demonstrates a high level of agreement with clinician-led detection methods [27–30]. Our previous Ghana-based studies confirmed HPVSS to be culturally acceptable [32–36] and effective in detecting precancerous lesions among WLWH in Ghana [35].

The WHO recommends HPVSS for use in LMICs [35, 37, 38]. However, despite its potential to increase screening uptake, its utilization is constrained by factors like insufficient awareness, perceived difficulty with self-sampling, and fear of positive results [39–41]. Effective communication is therefore needed to increase public engagement on HPVSS. To address this, we integrated the 3R communication model into HOPE [42, 43].

#### Evidence-based 3R communication model

The dimensions of the 3R communication model are reframing, reprioritizing, and reforming. Reframing is derived from health communication strategies [34, 35] that contrast the consequences of not screening and delayed treatment of precancerous lesions of the cervix (loss-framed appeals) with the benefits of early detection and treatment (gain-framed appeals). Reprioritizing prompts individuals to consider the relatively low cost of screening and treating precancerous lesions against the significantly higher cost of cancer treatment and possible loss of life. Reforming involves correcting misconceptions about self-sampling to promote positive attitudes toward screening and treatment. In Ghana, application of the 3R model within the HOPE toolkit significantly increased screening uptake and treatment compliance, achieving100% rates in the intervention group [36, 37].

#### Evidence-based patient navigator support

Patient navigators, trained lay or volunteer health workers, play a vital role in improving equitable access to, and sustained use of HPVSS [37]. In Ghana, they routinely deliver health education and community-based services for various conditions, including breast cancer and HIV [44]. Within the HOPE intervention, patient navigators motivated WLWH, facilitated questionnaire completion, addressed participant questions, and ensured timely distribution and return of HPVSS kits. These activities enhanced service uptake, improved screening rates, and reduced diagnostic delays, consistent with findings from other LMICs [23, 45, 46].

### Implementation science framework and applications

HOPE-inG applies implementation science methods to facilitate the adoption of HOPE’s evidence-based components into Ghana's healthcare system. The goal is to enhance the adoption, fidelity, and sustainability of these interventions, ensuring they are effectively embedded within existing health infrastructure and are responsive to local context and needs.

**Fig. 1:**
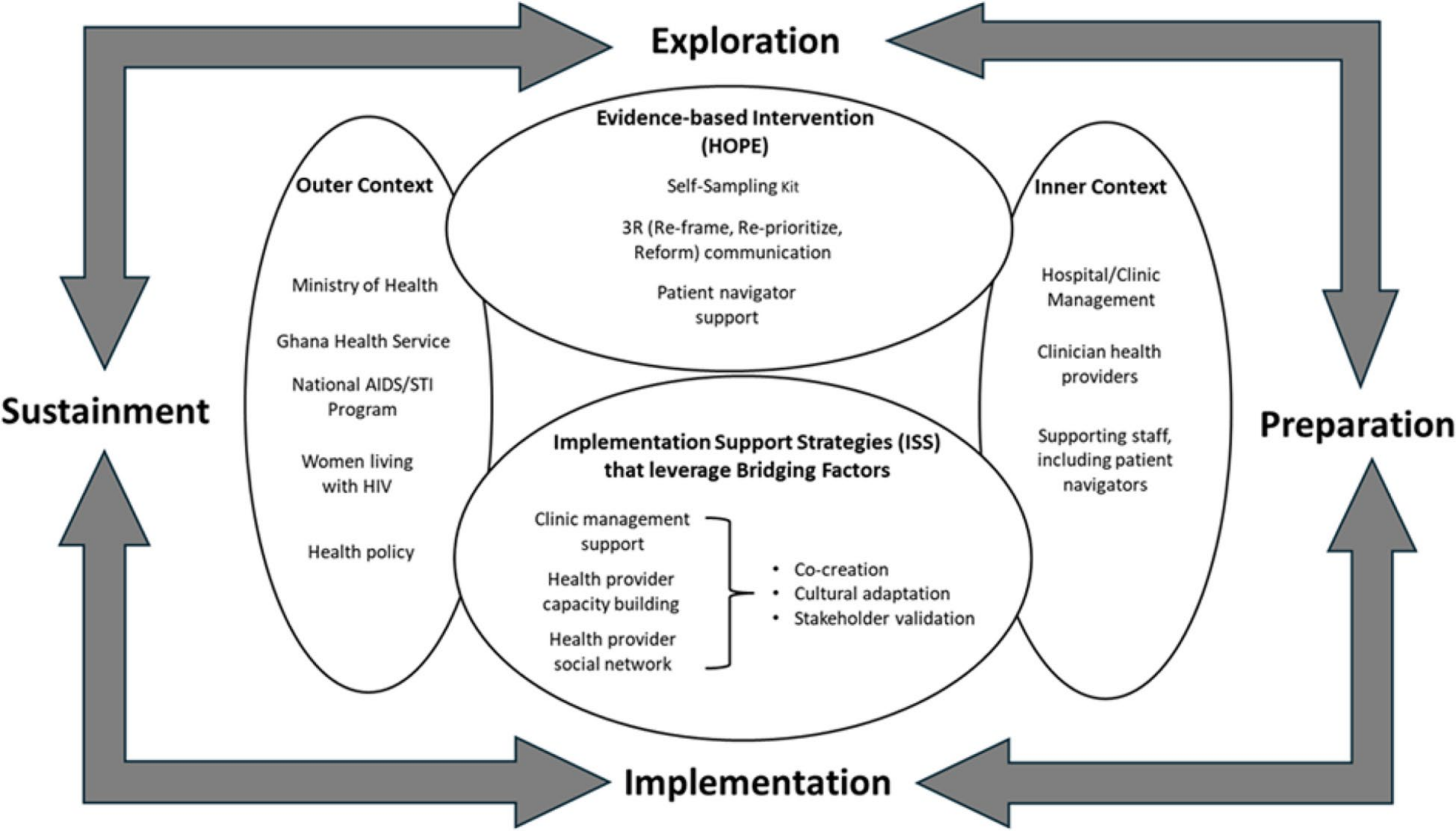
Conceptual framework for HOPE intervention implementation

We have selected the Exploration, Preparation, Implementation, and Sustainment (EPIS) implementation science framework [47, 48] to guide this project. EPIS is a combined determinant-process framework that is phased and multilevel and guides evidence-based interventions' planning and implementation process. Key constructs of EPIS are (1) the EBI(s), (2) Bridging factors, (3) Outer context, and (4) Inner contexts. EPIS focuses on identifying and leveraging bridging factors that connect the Outer and Inner contexts and is useful in guiding EBI implementation towards established practice in different settings, including in LMICs [48, 49] (Fig. 1.)

#### Bridging factors and implementation support strategies

Bridging factors are formal relationships and processes that exist between the Inner organizational context and the Outer policy/funding system context [50]. Bridging factors are complex but critical driving forces for EBI implementation and sustainment [50]. This proposal aims to translate the HOPE EBI into practice, and implementation strategies that specifically leverage bridging factors to achieve adoption, scale-up, and sustainment will be highly relevant. For the HOPE-inG study, we refer to implementation strategies that specifically leverage bridging factors as “implementation support strategies” (ISS), given that implementation support is technical and logistical assistance in implementing evidence-informed practices, policies, and programs, and in sustaining and scaling evidence for population impact [51]. (Fig. 1). In other words, HOPE-inG’s ISS will facilitate the routinization and integration of the HOPE EBI into practice by leveraging bridging factors that connect the Inner organizational and Outer funding/policy contexts.

#### The outer and inner contexts

The HOPE-inG Outer Context consists of policymakers (Ministry of Health, Ghana Health Services, National AIDS and Sexually Transmitted Infection Control Program (NACP)) and their policies (e.g., cervical screening, treatment, and insurance policies) (Fig. 1). The Ministry of Health formulates health policy and sets standards for healthcare. The Ghana Health Service oversees day-to-day health services and implements health policies. The NACP provides care and support services for persons with HIV. The Inner Context embodies the organizational clinic setting (study sites) and consists of clinicians implementing HOPE (e.g., physicians and nurses), supporting staff (e.g., patient navigators), and health facility administrative staff.

### Formative study in Preparation Phase

We conducted a formative study to identify barriers and facilitators to implementing and scaling up the HOPE intervention in Ghana’s health system. The findings informed the selection of potentially high-impact and feasible ISS. We used a nominal group technique (mixed methods) and the Consolidated Framework for Implementation Research (CFIR, qualitative) [52] to rapidly collect and organize data on barriers and facilitators from a total of 65 people in four nominal groups: WLWH (intervention recipients; *n* = 16), healthcare workers (intervention deliverers; *n* = 16), health facility managers (*n* = 16), and government officials (*n* = 17) (decision-makers). Participants generated their ideas individually and then shared and clarified them with the group in a round robin fashion. Thereafter, all ideas were ranked according to frequency, feasibility, and health system priority.

Here, we briefly report the formative study findings. The top three to five determinants from each nominal group were then collated into a priority list. A total of 29 highly ranked determinants (13 facilitators, 16 barriers) were identified across all five CFIR domains: Inner Setting (12), Outer Setting (6), Implementation Process (6), Innovation (2), and Individuals (4). These determinants were then matched to 73 implementation strategies from the Expert Recommendations for Implementing Change (ERIC) that were deemed feasible [53]. Out of the 73 matched implementation strategies, 24 were identified as ISS and relevant to implementation support in the Inner Setting and at the interface of the Inner and Outer Setting.

We used Lengnick-Hall et al.’s bridging factor “form and function” dimensions [50] to highlight key bridging factors and ISS identified for local implementation of the HOPE intervention, and ISS identified from the nominal groups that address these key bridging factors (Table 1).

**Table 1:** Prioritized bridging factors and implementation support strategies for HOPE implementation

Bridging Factor	Type	Capital Exchanged	Proposed Implementation Support Strategy	Impact on Inner and/or Outer Context
Advisory interactions between management and government health officials	Relational tie	Technical support from officials to management	Support program for clinic management to include government advice	Improved health facility management support and implementation climate for providers implementing HOPE
Government-approved policies and delivery modalities for screening and treatment	Process	Clinical and logistical guidance for providers' implementation of HOPE	Healthcare provider support and capacity building	Improved provider knowledge and self-efficacy in delivering HOPE. Pathway to sustained and structured HOPE implementation
Inter-agency collaboration in guiding and refining HOPE implementation	Relational tie	Social capital, sources of power involvement, existing infrastructure and resource alignment	Multiagency representation (e.g., SAB) for HOPE implementation	Synergy in government policy, existing resources and infrastructure for sustainable HOPE implementation at facilities
Government role in social network support for HOPE implementers	Relational tie Process	Implementation experiences Moral and technical support	WhatsApp^©^ group to foster social network support for providers implementing HOPE	Ongoing government, clinical (from Teaching Hospital) and peer support for providers implementing HOPE. Better insight into challenges and opportunities
Contribution of government/facility vs patient funding in HOPE intervention	Formal arrangement	Money	Discuss expectations and decision-making on funding arrangements for HOPE in SAB	Government and facility-level clarity, expectations set and concrete information shared on HOPE funding and degree of integration into routine care

Inner setting: health facility; Outer setting: government agencies, funding, policies, and procedures

Additional bridging factors may be identified, and ISS will be reviewed and refined during study implementation

At the end of the formative study, a ten-member Stakeholder Advisory Board (SAB) was convened among nominal group participants, comprising WLWH, healthcare providers, clinical managers, and policymakers. The Board provides high-level technical and contextual expertise in designing and implementing the intervention and in disseminating findings to the broader body of stakeholders in Ghana.

### Site selection

The proposed research will be conducted in the Central Region, home to approximately 2.8 million people [54] and has the fifth-highest HIV burden among Ghana’s 16 regions [55]. We will leverage established partnerships with HIV clinics in the Central Region and with the National AIDS/STI Control Program (NACP) to select secondary-level facilities with similar infrastructure. In the Central Region, 85 antiretroviral therapy (ART) clinics operate across 22 districts. The selection criteria included: a clinic that is the largest district-level hub site, has highly accessible spokes, and has enrolled at least 400 WLWH on ART. The largest hub sites that have already established access to cervical cancer screening and treatment services at a tertiary referral facility were excluded. A master list of study-eligible women was developed through the existing HIV E-tracker electronic medical record system. Ultimately, four study sites were selected for randomization for the intervention arm (two sites: Our Lady of Grace Hospital, Breman Asikuma and Saltpond Municipal Hospital, Mfantseman) and the control arm (two sites: Saint Francis Xavier Hospital, Assin Fosu and Swedru Government Hospital, Agona West). 

The combined workforce across the selected sites includes approximately 500 staff and a total bed capacity of over 700. For project implementation, we will engage existing personnel, including health facility administrators, clinicians (doctors, nurses, midwives), and support staff (including patient navigators), collectively referred to as “healthcare providers”.

### Study Preparation, Implementation and Sustainment Phases

We have completed the Exploration Phase, where we evaluated cervical screening needs and the fit of the HOPE intervention in Ghana. Through prior efficacy, formative, and effectiveness studies, we identified WLWH and healthcare workers' needs for cervical screening [23, 35, 56, 57], which guided our development and evaluation plan for HOPE. HOPE-inG thus focuses on EPIS’ remaining three phases for the five-year study period:

- Preparation Phase (Year 1): relevant to Aim 1, where we identified potential barriers and facilitators of HOPE implementation and developed a health system implementation plan and provider’s training content for HOPE implementation. This phase has been completed.
- Implementation Phase (Years 2 and 3): relevant to Aim 2, where we assess the effectiveness of the HOPE 2.0 intervention and the success of the implementation plan.
- Sustainment Phase (Years 3–5): relevant to Aim 3, where we assess the impact of the implementation plan on the sustainment of HOPE 2.0 implementation at study sites.

Study design, population, analysis, and outcome measures are described per study phase.

### Preparation phase

To develop a culturally appropriate, evidence-based implementation plan and provider training materials to support the effective delivery of the HOPE 2.0 intervention within Ghana’s health system.

#### Design

We are applying an explanatory sequential mixed methods design to develop and refine a plan for integrating the HOPE 2.0 intervention into Ghana’s existing health system. We have already completed the nominal groups for this phase, with identification of determinants, relevant implementation strategies, and ISS, as described above. The next steps will be to apply these formative findings to modify HOPE to HOPE 2.0, along with the development of a HOPE 2.0 health system implementation plan and provider implementation training materials relevant to the plan.

#### Study population

Besides the 65 participants from the nominal groups already described, we plan to purposively select and survey 10 providers from each study site (total *N* = 40). This pre-implementation baseline survey will focus on implementation outcomes of feasibility, acceptability, and appropriateness of the HOPE 2.0 implementation plan (including HOPE 2.0 and ISS), using the psychometric scales developed by Weiner et al. [58] Findings from this survey will be used to refine the implementation plan.

#### Outcome measures and analysis

Nominal group analysis has already been described above. We will use descriptive statistics recommended by Weiner et al. [58] to analyze the feasibility, acceptability, and appropriateness data. These measures are brief and each takes about 5 min to complete, comprising four questions posed to the participant with responses to each question provided on a five-point Likert scale: *Completely disagree*; *Disagree; Neither agree nor disagree*; *Agree*; or *Completely agree.* Individual participant scores will be computed for each scale with a maximum score of 20 per scale (max 5 points for each of 4 questions), with the mean computed across participant groups. We will then categorize the individual scores in each participant group into tertiles, as high, middle, and low scores for each scale.

### Implementation phase

Assess the effectiveness of the HOPE 2.0 intervention and the success of the implementation plan in a hybrid type 2 trial.

*Hypothesis: Cervical screening uptake by HPVSS will be significantly higher among WLWH in the intervention vs. the control group, and implementation outcomes scores among intervention group providers will be significantly higher than those of control group providers.*

#### Design

We will conduct a hybrid type 2 cluster randomized controlled trial to simultaneously evaluate the effectiveness and implementation of HOPE 2.0 using bridging factor-focused ISS. HIV clinics at the four selected secondary-level facilities will be cluster randomized 1:1 in a two-arm trial. WLWH in the intervention group will receive HOPE 2.0 from providers trained and supported to implement HOPE 2.0 using ISS-focused training materials and per the implementation plan developed in the Preparation Phase. After this intensive 6 month implementation phase, technical and clinical support from the study team and clinical experts to the intervention sites will be withdrawn, after which the Sustainment Phase will begin

**Fig. 2:**
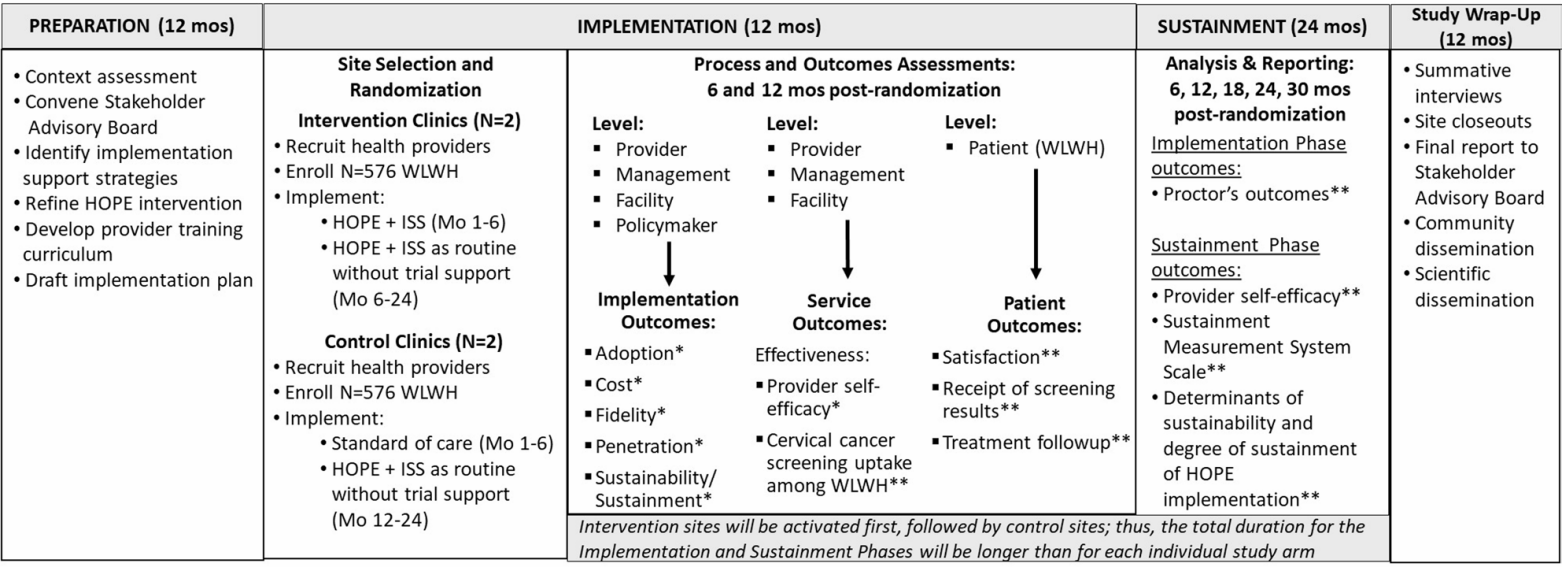
Study implementation procedures and process/outcome assessment time points. WLWH: women living with HIV; ISS: implementation support strategies

WLWH in the control group will also receive HOPE, from providers without ISS or an implementation plan (Table 2 and Fig. 2). At the end of this 6 months period of implementation, the study team will provide ISS materials to control group health facilities to co-implement with HOPE 2.0. There will be a similar Sustainment Phase for the control sites.

**Table 2:** HOPE-inG baseline provider training in intervention vs control arms

	**Topic**	**Intervention Arm**	**Control Arm**
1	Epidemiology of cervical cancer (CC) in women with and without HIV	Yes	Yes
2	The WHO cervical cancer elimination agenda	Yes	Yes
3	The HOPE intervention: components and evidence	Yes	Yes
4	Cervical cancer screening strategies	Yes	Yes
5	Pre- and post cervical screening counselling	Yes	Yes
6	Psycho-social support for clients with positive cervical screening results and their immediate family	Yes	Yes
7	Awareness creation, record keeping and documentation	Yes	Yes
8	Research ethics: participant recruitment, study procedures and collecting, documenting and reporting data	Yes	Yes
9	Delivering the HOPE intervention in Ghana: Implementation research to practice	Yes	No
10	Who is supposed to do what, where and when? The HOPE 2.0 implementation plan	Yes	No
11	How will we deliver the intervention? Implementation support strategies for HOPE 2.0	Yes	No
12	What resources do we have for successful implementation? Additional tools, job aids, support and training for delivering HOPE 2.0	Yes	No

#### Study population

We will recruit participants representing four organizational levels, including WLWH patients, healthcare providers, clinic managers, and policymakers.

### Inclusion and exclusion criteria and recruitment

#### Inclusion criteria

General eligibility criteria are living and/or working in the Central Region and ≥ 18 years old.

- Specific eligibility criteria for providers are to possess healthcare training and/or qualifications (i.e., patient navigators, physicians, nurses) and work at the HIV clinic; for clinic managers, health facility management in administrative leadership, and oversee HIV clinic operations.
- Study eligibility criteria for women in the trial include women who are living with HIV (regardless of pregnancy status), between 25 and 65 years old (age consistent with WHO cervical screening guidelines [2]); have never had cervical screening (Pap smear or HPV test), or have not had cervical screening in the last five years.
- Eligible community members include individuals (with and without HIV and/or cervical cancer) living in the study communities and/or family members of WLWH.
- Eligible policymakers include personnel from the National AIDS/STI Control Program, Ministry of Health, and Ghana Health Service.

#### Exclusion criteria

WLWH who have a cervix are the main target population for the HOPE-inG study. WLWH who or have had a total hysterectomy (inclusive of the cervix) will be excluded.

#### Recruitment

- *Doctors, nurses, patient navigators, and clinic managers:* We will meet with Medical Directors of the selected health facilities to discuss the study and the recruitment of providers. We will recruit providers by email, phone, and face-to-face contact.
- *WLWH* will be recruited using E-tracker electronic records. Participating providers at each site will identify eligible women from their site-level sampling frame (400–600 per site for a total pool of ~ 3,900 WLWH) and contact them by phone to solicit participation. Verbal informed consent will be sought in English/applicable local language by research coordinators prior to trial initiation. Participant recruitment is ongoing and will be completed by March 2028.
- *Community members* will be recruited from study communities via community gatekeepers.
- Ghana Health Service and the Ministry of Health will facilitate the recruitment of government officials through written and verbal invitations and snowball recruitment.

#### Sample size estimation

The sample size is calculated based on implementation outcomes of health system adoption, cost, fidelity, penetration, and sustainability.

- *For power calculation*, we used Power (1 − β) = 0.8 and α = 0.05 and did not assume equal cluster sizes. The coefficient of variation (CV) of cluster sizes is set to 0.2, allowing for a large variability between clusters. Methods from Donner et al. [59] implemented in R for cluster RCTs were used.
- *For binary outcomes,* e.g., adoption, we assumed 15% adoption in the control group based on previous study [23]. Table 3 shows the sample size needed to detect increases in implementation outcomes (e.g., adoption) in the intervention arm from 15% to 25–50% with 80% power. We expect to achieve an increase of at least 15%, to 30% or more, for outcomes such as adoption.
- *For continuous outcomes* such as sustainability/sustainment, we used estimated variance = 1 (standardized), and variance within clusters = 0.95 (assuming a large proportion of individual WLWH variability). For average cluster sizes (sampling frame per facility) ranging from 400 to 600 WLWH, we have greater than 80% power to detect differences in mean scores between the intervention and control arms as small as 1.4. We aim to recruit at least 1,152 patients (288 per site × 4 sites, inclusive of 20% attrition for a minimum sample size of 240 per site) Thus, the sample size for the Intervention Arm is *n* = 576 and Control Arm is *n* = 576.

**Table 3:** Power and sample size calculation

Intervention Numeric	Control Numeric	Minimum Sample Size Per Site
0.25	0.15	504
0.30	0.15	240
0.35	0.15	142
0.40	0.15	94
0.45	0.15	67
0.50	0.15	50

### Randomization

**Fig. 3:**
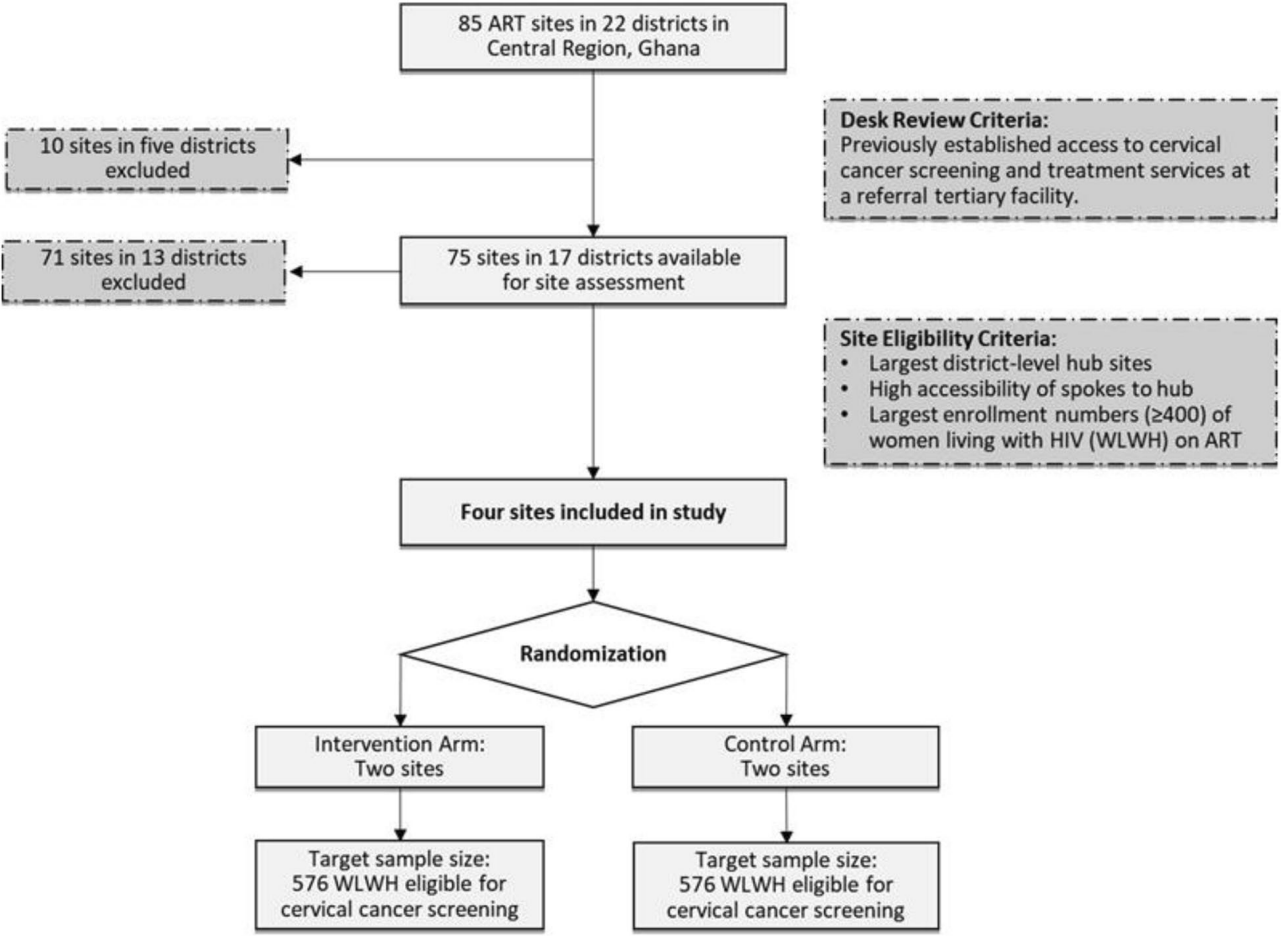
Flow diagram for HOPE-inG study site selection

Study sites are the unit of randomization, and all providers and WLWH at a given HIV clinic at each site will be randomized to the same study arm. Using computer-generated numbers with a block of 2, the four study sites will be randomized to one of two arms: two sites in the intervention group and two sites in the control group (Fig. 3).

### Study procedures

#### Intervention arm

**Fig. 4:**
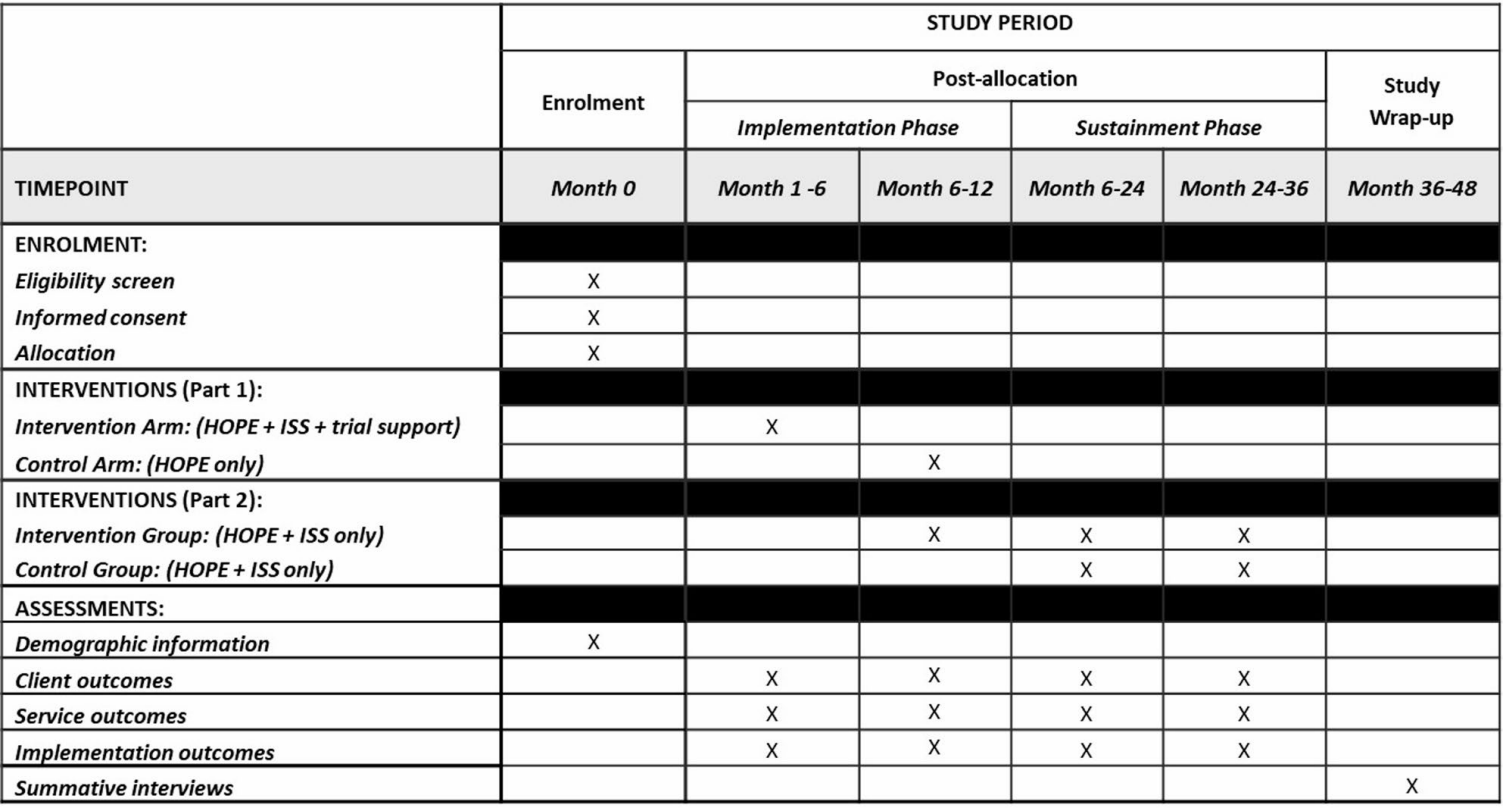
Schedule of enrollment, interventions, and assessments. ISS: implementation support strategies

- Step 1. Providers, i.e., clinicians, patient navigators, and clinic management at intervention sites will receive a) ISS delivered through management support, capacity-building, and social network support, and b) baseline and refresher training on the HOPE implementation plan.
- Step 2. Clinicians will a) screen and recruit eligible WLWH, and b) implement HOPE 2.0 by delivering 3R messages via WhatsApp to enroll WLWH.
- Step 3: WLWH will receive a self-sampling kit from clinicians or patient navigators to collect samples.
- Step 4: WLWH with positive screening tests will follow up for treatment per the WHO screen, triage, and treat cascade (Fig. 4). Patient navigators will support participants to follow up.

#### Control arm

Providers and clinic managers at control sites will receive baseline training on study implementation, but will not receive any ISS or implementation plan training (Table 2). They will, however, be required to follow Steps 2, 3, and 4 described in the intervention arm described above, except for patient navigator support.

#### Assessments

Program implementation assessments for HOPE 2.0 will be conducted at multiple organizational levels using mixed methods [60] (Table 3).

Multiple points of assessment allow us to determine the progression of implementation and outcomes at different organizational/policy levels. Surveys will be completed in person or online via Qualtrics platform links and/or QR codes sent by SMS and email. Healthcare workers and research assistants will help WLWH complete the in person and online surveys. The standard of post-trial care is provided to research participants through Cape Coast Teaching Hospital. No compensation is available to participants who suffer harm from trial participation.

### Outcomes measures

The primary outcomes of interest in this type 2 hybrid trial are 1) the effectiveness of the HOPE 2.0 intervention in increasing uptake of cervical screening among WLWH, and 2) the adoption of HOPE 2.0 intervention at the facility level. Other outcomes, including cost, fidelity, penetration, and sustainability, will be assessed as secondary outcomes. Additionally, Proctor et al.’s [61] client (i.e., WLWH) health outcomes will also be assessed (Fig. 2).

- *Cervical screening uptake* is defined as the proportion of WLWH in each study arm that successfully complete the cervical screening process. In the intervention arm, completing the process requires the WLWH to present to the clinic, collect a self-test kit, collect a cervical sample, and submit the sample to the clinic for testing. In the control arm, completing the process requires the WLWH to present to the clinic and receive a pap smear/cervical sample collection from a provider. WLWH in either study arm who do not complete the entire process will not be considered as completing uptake. Uptake assessment will therefore be determined as proportion of WLWH with complete screening out of all women enrolled in each study arm.
- *Program Adoption* is defined as the decision of healthcare workers (i.e., providers and management) and patients (described as patient uptake) to commit to and use HOPE 2.0 and ISS adoption. Adoption will be measured at provider, management, and patient levels using a variety of data logs, surveys, and interviews.
  - At the management level, we will assess (i) the number of ISS training sessions attended and (ii) the degree to which ISS components are used to support providers.
  - At the provider level, we will assess (i) the number of ISS training sessions attended, (ii) the degree to which ISS components are used, and (iii) the number of implementation plan steps followed. Additionally, we will assess providers’ (i) implementation experience (i.e., resource availability, managerial support, and technical issues confronted); and (ii) operational experience (i.e., workload increase or decrease, flow of health facility communication, and any skills needed).
- *Implementation Cost* is defined as the total expenses (facility/provider and patient) for implementing HOPE 2.0. Cost will be assessed at the health facility level in both study arms. We will use a micro-costing approach to estimate the economic costs associated with each study arm. The direct measure method of micro-costing involves tracking resources used and enumerating unit costs for each resource to precisely estimate cumulative costs associated with each study arm [62]. Implementation cost will be measured at the health facility level and patient level, utilizing a variety of cost-tracking databases throughout the study period to itemize, quantify, and value resources.
  - At the health facility level, a cost-tracking database will be used throughout the study period to itemize, quantify, and value resources. Costs at the facility level include (i) number of hours staff spent on HOPE 2.0 training; (ii) sampling materials, including kits and laboratory testing costs; (iii) recruitment (i.e., time spent identifying and contacting eligible women, advertising materials, participant incentives); (iv) HOPE 2.0 delivery (with and without ISS); and (v) treatment and biopsies.
  - Cost to the patient will include tracking of participant out-of-pocket costs (e.g., transportation, childcare, phone data, time spent on HOPE 2.0 procedures). French et al.’s outpatient cost survey [63] (5 items) will be adapted to measure patient-level costs.Note: Time effort will be estimated using the hourly wages of WLWH and healthcare workers.
- *Fidelity* is the degree to which HOPE 2.0 and its ISS are implemented as planned.
  - At the provider level, utilizing surveys, log sheets, and interviews, we will assess (i) the degree of adherence to HOPE 2.0/ISS protocols (Intervention) and HOPE 2.0 (Control) during implementation, (ii) the dose of the program delivered, and (iii) the quality of program delivery.
  - At the management level, we will assess (i) adherence to ISS protocol (Intervention only) and (ii) overall management support for providers (both arms).
  - We will utilize log sheets and interviews, as well as observations, and the Process Evaluation Checklist (PEC) [64] throughout to track the fidelity of implementation and overall project progress.
- *Program Penetration* for this proposal is the integration of HOPE 2.0/ISS within the HIV subsystem of study facility settings [61]. We will assess penetration outcomes at provider and management levels by determining the proportion of personnel who actually deliver on a HOPE 2.0 program task/role, out of the number trained or expected to deliver on that task/role.
  - At the management level, we will utilize log sheets and interviews to measure the expected tasks or roles to be evaluated, which will be (i) the provision of technical support and (ii) facilitating a conducive HOPE 2.0 implementation climate for providers.
  - At the provider level, we will utilize log sheets and interviews to measure the expected tasks to be evaluated, which are (i) screening and recruitment of eligible WLWH, (ii) delivery of HOPE 2.0 to enrolled WLWH, and (iii) follow-up on all testing outcomes and completion of required documentation for these tasks/roles.
- *Program Effectiveness* is defined as the impact of the ISS on 1) providers' self-efficacy in implementing HOPE 2.0, 2) WLWH screening uptake, and 3) treatment outcome. We will assess effectiveness outcomes at management, provider, and WLWH levels utilizing surveys, log sheets, and interviews.
  - At the management level, we will assess (i) the frequency of support that management delivers to providers, (ii) management self-efficacy in supporting HOPE 2.0 implementation, and (iii) challenges in supporting HOPE 2.0 implementation at their facilities, utilizing log sheets and interviews.
  - At the provider level, we will assess (i) self-efficacy for implementing HOPE, (ii) fidelity of HOPE 2.0 intervention delivery, (iii) number of WLWH screened and women with positive results who are treated, and (iv) the type, frequency, duration, and quality of service provided to patients (WLWH).
  - At the WLWH level, we will assess the impact of HOPE 2.0 on patients’ (WLWH) screening uptake (i.e., service outcome) and receipt of screening results, patient satisfaction, and for women with positive screening results, clinical follow-up, and evaluation for, and receipt of CC treatment where appropriate (i.e., client outcomes). Assessment procedures: WLWH in both study arms will complete the screening and satisfaction outcome survey within one month of completing HOPE 2.0. Within one month of screening, providers will check the lab registry and will review the electronic medical record to confirm screening completion. Treatment compliance will be evaluated within 6 months of positive result notification. The screening and treatment completion outcomes will be binary (yes/no), test results (positive, negative, or inadequate), treatment follow-up (yes, no, or lost-to-follow-up), and patient satisfaction will be measured using a patient satisfaction survey [65].

### Qualitative data collection

Following completion of the 6-month surveys at the intervention sites, focus group discussions and key informant interviews will be conducted at four organizational levels (i.e., patient, provider, management, policy) for an in-depth understanding of contextual factors influencing the implementation outcomes of interest. At least one patient focus group and three key informant interviews representing providers and managers will be conducted at each study site with 1 to 2 key informant interviews relevant to the district or the Central Region. The target numbers of participants indicated are estimates; the actual numbers for each study site will be determined when saturation is reached [66]. Interviews will be conducted by trained research assistants supervised by the researchers. We will use a semi-structured interview guide [67] to conduct 60-min audio-recorded interviews.

### Statistical analysis plan

Data will be cleaned and reviewed to determine if distributional assumptions are met. If not, scores will be transformed, or appropriate nonparametric methods will be applied. If distributional assumptions are valid, sensitivity analyses (with and without outliers) will be applied. Hypothesis testing will use α = 0.05 (2-tailed). An intent-to-treat analysis will be used to maintain baseline comparability achieved by randomization.

#### Quantitative data analysis

- Descriptive analysis will be used for implementation outcomes scores at each health facility; scores will be compared within health facilities and between study arms over time. Mixed models (Linear Mixed Models or Generalized Linear Mixed Models) will account for repeated participant measures.
- *For Likert scale items (surveys)*, we will use ANOVA to determine differences in implementation outcome scores between study arms, with Tukey HSD (honestly significant difference) post hoc analysis to determine specific sites that significantly differ.
- *For continuous outcome variables*, we will similarly use linear mixed models to analyze treatment effects while controlling covariates, including age, education, income, religion, marital status, employment, insurance, and clinical conditions consistent with our previous studies [68, 69].
- *Fidelity analysis:* The fidelity checklist and activity log sheets at each study site will be aggregated, and composite site scores will be calculated and compared across intervention and control sites.
- *Cost-Effectiveness Analysis:* Costs will be determined using the cost trackers described. At the facility level, we will use the WHO Incremental Cost-Effectiveness Ratio (ICER) [70]. A formula to analyze the cost-effectiveness outcome, whose outcome of interest is screening and treatment. An ICER is calculated by dividing the difference in total costs (incremental cost) by the difference in the chosen measure of health outcome or effect (incremental effect) to provide a ratio of ‘extra cost per extra unit of health effect. We will determine the Total Cost [average fixed and variable costs (TC)] for screening and treatment in IG (Arm 1) and CG (Arm 2), and cervical cancer cases averted (incremental effect) for each Arm. Then ICER = Incremental cost/Incremental effect. An ICER of less than 1 indicates that the control group is cost-effective, an ICER greater than 1 suggests the intervention group is cost-effective, and an ICER equal to 1 indicates a similar cost for both arms. At the patient level, we will calculate the average cost per patient in both arms.

#### Qualitative data analysis

Recorded audio interviews will be transcribed verbatim; transcripts in local languages will be translated into English before analysis. Qualitative analyses will be conducted at WLWH, provider, management, and policymaker levels. We will use NVivo to manage all qualitative analyses for all Aims, via thematic coding and content analysis [71]. Two coders will independently read transcripts and identify common themes. Cohen’s kappa < 0.70 (intercoder reliability) [72] will be discussed, coders will decide on a final coding scheme [73], and data will be summarized to determine how themes are interrelated.

#### Mixed methods analyses

We will use an explanatory sequential design [74]. Survey data will be analyzed, followed by interview data. After analysis, we will integrate both data, develop a table (joint display) to show how the QUAL results enhance QUANT results, and interpret the value added by qualitative explanations.

### Sustainment phase

Assess the impact of the implementation plan on the sustainment of the HOPE 2.0 intervention at study sites.

*Hypothesis: The deployment of culturally adapted, bridging factor-focused ISS will significantly increase the sustainment of HOPE 2.0 at HIV clinics in the IG compared to the CG.*

#### Study design

We will use a mixed-method design to assess the sustainment of the program. Sustainment is the extent to which the intervention (HOPE 2.0 ± ISS) is integrated within the HIV clinics’ routine operations [47, 48, 61].

#### Study population

From 6 to 18/24 months post-randomization for both intervention and control sites, we will measure determinants of sustainability and degree of sustainment at the provider (total *n* = 6—10), management (*n* = 2—4), and policymaker (*n* = 2-4) levels, using the Sustainment Measurement System Scale (SMSS) [75], (Fig. 4, Table 4).

**Table 4:** Domains of determinants and the degree of sustainability in the Sustainment Measurement System Scale

Domains	Data to be Collected*
1. Financial stability	Surveys and interviews
2. Responsiveness to community needs	Surveys and interviews
3. Responsiveness to community values	Surveys and interviews
4. Partnerships	Surveys and interviews
5. Facility capacity	Surveys and interviews
6. Facility capability	Surveys and interviews
7. Implementation leadership	Surveys and interviews
8. Evaluation, feedback, outcomes	Surveys and interviews

^*^Survey data will be collected at baseline 6, 24, 12, 18, and 24 months post-randomization. Interview data will be collected at 6 months post-randomization

#### Analysis

For the quantitative and qualitative analyses for the sustainment phase, we will use the same statistical analyses described in Phase 2.

#### Outcome measures

At 6, 12, 18, 24-months post-implementation phase, we will assess sustainment at intervention and control sites using the SMSS scale (Table 5 shows measures of determinants of sustainability). This quantitative scale assesses the process of achieving sustainment in eight domains of sustainability determinants: (i) financial stability; (ii) responsiveness to community needs; (iii) responsiveness to community values; (iv) coalitions, partnerships, and networks; (v) health facility capacity; (vi) health facility staff capability; (vii) implementation leadership; and (viii) evaluation, feedback, and positive program outcomes. We will further evaluate sustainment by including qualitative questions for the eight SMSS determinants in previously described interviews at specific time points, including in the 6-month post-trial period (Fig. 4, Table 4). At the end of the HOPE 2.0 trial, we will provide ISS materials to control sites to co-implement with HOPE 2.0. At 6, 12, 18, 24 intervention phase.

## Discussion

The HOPE-inG study evaluates the implementation and sustainment of the HOPE intervention, a multicomponent cervical cancer prevention strategy tailored to meet the unique needs of WLWH in Ghana. Grounded in implementation science and guided by the EPIS framework [47, 76]. HOPE-inG integrates evidence-based components, including HPV self-sampling, the 3R communication model, and patient navigation support, within a context-sensitive implementation plan. The intervention and ISS were co-developed with key stakeholders to ensure cultural congruence, systems alignment, and community responsiveness.

The Preparation Phase was central to ensuring contextual fit and long-term sustainment. This participatory approach fostered early stakeholder buy-in and increased the relevance, feasibility, and acceptability of the intervention, factors known to be critical for successful implementation in low-resource settings [77–79].

We are now at the beginning of the Implementation Phase, where we will simultaneously assess effectiveness and implementation outcomes [80, 81].

### Comparison to prior work

While there is an ample body of evidence from high income countries, few studies have rigorously investigated the effectiveness of HPV self-sampling in increasing cervical screening uptake in LMICs [82–85]. Notable studies in Africa include 1) the ongoing CHESS trial evaluating both uptake and sustainability of HPV self-sampling among WLWH in Nigeria [86]; 2) a home-based vs clinic-based HPV self-sampling trial on uptake among WLWH in Uganda [87]; and 3) the programmatic CarciSCAN HPV self-sampling study on uptake among women in Ghana's general population [88]. The HOPEinG study focuses on both effectiveness and long-term sustainability and will be well positioned to narrow the evidence gap for HPV self-sampling in cervical cancer control within Ghana and across Africa.

#### Anticipated challenges and mitigation strategies

First, cross-contamination between intervention and control arms could bias results; to mitigate this, we adopted a cluster randomized design with geographically dispersed sites and will apply Complier Average Causal Effect (CACE) analyses if contamination occurs. Second, missing data may affect validity; we accounted for anticipated attrition, will use multiple imputation under a Missing at Random (MAR) assumption, and conduct sensitivity analyses for Missing Not at Random (MNAR) scenarios. Third, recruitment challenges may arise despite prior success, so outreach will be expanded, networks leveraged, and new sites added if needed. Fourth, smartphone access is essential; loaner devices will be provided. Lastly, treatment costs for uninsured participants will be covered to ensure equitable participation.

##### Strengths and expected impact

The strengths of the HOPEinG study lie in its offer of a scalable, context-sensitive model for integrating cervical cancer prevention for WLWH into HIV services. HOPE-inG tackles structural, sociocultural, and systemic barriers by embedding cervical screening within trusted HIV care settings and applying locally-responsive implementation strategies that are focused on implementation support. The trial design promotes health system ownership and allows for ample time to study factors related to long-term sustainment. Its mixed-methods design generates rich insights into multilevel barriers and facilitators, informing broader policy and program decisions in resource-limited settings. The study advances implementation science by providing empirical evidence on ISS for increasing cervical screening uptake in low-resource settings. Ultimately, findings will inform national policy for cervical cancer prevention and for patient-driven and community-based screening programs in similar settings across Africa.

## Conclusion

The HOPE-inG intervention provides a robust, scalable model for embedding cervical cancer prevention into HIV care systems in low-resource settings. The integration of combination evidence-based tools with stakeholder-driven implementation support strategies leverages the science and advances the practice of implementation in LMICs. The findings have broad implications for how health systems can sustainably novel non-communicable disease care models into established health programs for populations most at risk.

## Supplementary Information

Supplementary Material 1.

## Data Availability

In line with NIH data sharing policies, we are committed to sharing our study results with everyone involved, participants, the general public, and the research community. Once the main findings are published, a de-identified version of the final dataset will be available to qualified researchers upon request, following NIH guidelines. We will also provide clear documentation, including a description of the study, details about how data were collected and analyzed, and a data dictionary, to help others use the information responsibly and build on our work.
